# Occupational Health and Safety of Immigrant Workers in Italy and Spain: A Scoping Review

**DOI:** 10.3390/ijerph16224416

**Published:** 2019-11-11

**Authors:** Cecilia Arici, Elena Ronda-Pérez, Tishad Tamhid, Katsiaryna Absekava, Stefano Porru

**Affiliations:** 1 Department of Diagnostics and Public Health, Section of Occupational Health, University of Verona, 37134 Verona, Italy; stefano.porru@univr.it; 2 University Research Center “Integrated Models for Prevention and Protection in Environmental and Occupational Health”, Universities of Verona, Brescia and Milano Bicocca, 37134 Verona, Italy; 3 Public Health Research Group, University of Alicante, San Vicente del Raspeig, 03690 Alicante, Spain; elena.ronda@ua.es; 4 Spanish Consortium for Research on Epidemiology and Public Health (CIBERESP), 28029 Madrid, Spain; 5 Postgraduate School of Occupational Medicine, University of Verona, 37134 Verona, Italy; tishad.tamhid@univr.it (T.T.); katsiaryna.absekava@univr.it (K.A.)

**Keywords:** migrant workers, occupational health and safety, occupational diseases, occupational injuries, economic crisis, working conditions, review, Italy, Spain

## Abstract

The main aim of the present study was to summarize the available literature on the topic of occupational health and safety (OH&S) among immigrant workers (IMWs) in Italy and Spain. We conducted a scoping review, searching Medline, Social Sciences Citation Index, PsycINFO, CINAHL Plus, SciELO, and EMBASE for peer-reviewed articles, published in English, Italian, or Spanish, between 1999–2018. 34 studies were included, 28 with quantitative methodology and 6 with qualitative. Main findings were that, compared to natives, IMWs in Italy and Spain showed higher prevalence of low-skilled jobs and of perceived discrimination at work; higher physical demands, poorer environmental working conditions, and more exposure to occupational risks (e.g., ergonomic and psychosocial hazards); a greater risk of occupational injuries; worse general and mental health; and a plausible worsening of their health status, especially in Spain, as a result of the economic crisis. The findings of the present scoping review constitute warning signs that indicate the need for a holistic global response to ensure that adverse OH&S outcomes among IMWs workers are improved and that equitable access to health care is guaranteed. Such a response will require a concrete and evidence-based approach to prevent and monitor occupational risk factors and associated outcomes in the workplaces.

## 1. Introduction

According to the International Labour Organization (ILO), there are 164 million migrant workers throughout the world; 95.7 million are men and 68.1 million are women. These migrant workers (i.e., international migrants, currently employed or unemployed, and seeking employment in their present country of residence) constitute 4.7% of all workers and present a high labour force participation, particularly in Europe [1]. It has been estimated that international migrants represent almost 10% (90.7 million) of the population in the 53 countries of the World Health Organization (WHO) European Region and that around 12% of all workers in this Region are migrants [2].

Due to their geographical position, Italy and Spain have played a significant role in the European migration crisis, being a logical passage for maritime arrivals who intend to find work in Europe [3,4]. Thus, both in Italy and Spain, there are about 6 million international migrants, representing 10% and 12.8% of the national population, respectively [2].

Immigrant workers, also because of language and cultural barriers, usually perform manual, unqualified, precarious, and low-paid jobs that natives tend not to perform anymore. They also work for longer hours and in worse conditions than do non-migrants. Moreover, they are more represented in the so-called “3D jobs” (dangerous, dirty, and demanding/degrading). Therefore, they have higher rates of adverse occupational exposures than natives, which lead to poorer health outcomes and higher risk of both occupational injuries and illnesses [1,5,6].

In Europe, most of the immigrant workers are employed in jobs entailing exposure to heavy physical work, risk of injury, exposure to toxic substances, or poor psychosocial working conditions (e.g., high mental workload, bad social interactions), which can negatively affect their health [7]. Hence, the occupational health and safety of immigrant workers is a very relevant aspect of public health in the European region [8].

Previous studies [3,4,9,10] showed that, compared to natives, migrant workers both in Italy and Spain

1. were more often hired on precarious contracts and mainly employed in manual, low-skilled jobs;
2. were concentrated in the most dangerous jobs;
3. presented a higher risk of occupational injuries and diseases; and
4. experienced a worsening of their working conditions during the international economic crisis of 2008–2014.

As in the rest of the world, there is an increasing female presence among immigrant workers, boosted by a large demand for often private and informal domestic workers and caregivers, likely a consequence of a shift of the burden of elderly and child care on the families and without full access to social protection and rights [6].

The aims of this study were

1. to summarize the available literature through a scoping review;
2. to identify research gaps; and
3. to make recommendations for future research on occupational health and safety among immigrant workers in Italy and Spain.

We decided to focus on these two countries for the following reasons:

1. previous systematic and scoping reviews on the topic of migrant workers’ health and safety did not cover specifically this geographic area [7,9,11,12,13]; and
2. these two countries are characterized by a similar immigration regimen—both encountered a deep economic crisis during 2008–2014 [3,4,14].

The crisis, in fact, changed the economic and employment prospects of immigrants in Italy and Spain by affecting the degree of labor market competition with native workers, especially for temporary and precarious jobs in the service sector. This situation forced many immigrants to leave the host country [3,4].

## 2. Methods 

We conducted a scoping review using the methods reported in the literature [7,9,15,16,17].

### 2.1. Search Strategy 

From October 2018 to March 2019, we searched Medline (through PubMed), Social Sciences Citation Index, PsycINFO, CINAHL Plus, SciELO Citation Index, and EMBASE databases. We searched for articles published in 1998–2018 in order to see how the occupational health and safety of immigrant workers in Italy and Spain developed over time, particularly before, during, and after the international economic crisis of 2008–2014.

We searched on terms related to immigration, then on terms related to occupational health and safety, and then related to the country of immigration:

String 1: Immigration descriptors

“Emigrants and Immigrants” OR “Emigration and Immigration” OR “Transients and Migrants” OR “Ethnic Groups” OR “Minority Groups” OR “Labour migrants” OR “Migrant workers” OR “Immigrant workers”

String 2: Occupational health and safety descriptors

“Work” OR “Employment” OR “Job” OR “Workload” OR “Occupational Exposure” OR “Occupational Risk” OR “Occupational Hazard” OR “Occupational Health” OR “Occupational Safety” OR “Occupational Medicine” OR “Occupational Diseases” OR “Occupational Accidents” OR “Accidents, Occupational” OR “Occupational injuries” OR “Economic recession” OR “Economic crisis” OR “Financial crisis”

String 3: Receiving country

“Europe” OR “European” OR “Italy” OR “Italian” OR “Spain” OR “Spanish”. 

Other potentially relevant sources were searched for in the Medline (through PubMed) database, starting from a reference list provided by the Editorial Office of the International Journal of Environmental Research and Public Health in July 2018.

### 2.2. Inclusion/Exclusion Criteria and Assessment

Titles and abstracts were primarily screened by three of the authors, to exclude those not meeting the following inclusion criteria: original articles published in peer-reviewed scientific journals (i.e., systematic and non-systematic literature reviews, commentaries, editorials, letters, conference abstracts or proceedings, theses, dissertations, books or book chapters were excluded), published in English or Italian or Spanish, mentioning immigrant populations and occupational health or occupational safety as central issues, and focused on immigrants (from any country of origin) employed in Italy and/or Spain.

Then, all potentially relevant papers were read in full by the first author, and a second selection was performed, applying the following inclusion criteria: papers reporting qualitative or quantitative studies addressing (a) the relationship between migration, work, and health; (b) occupational health and safety among immigrant workers; (c) the health status of immigrant workers or (d) preventive programs/activities performed among employed immigrants; (e) social, occupational, cultural, and/or economic determinants of health and safety among immigrant workers; or (f) access to and use of health services among immigrant workers.

Finally, each of the included articles was assessed by one of the authors and then the first author, using a set of predefined parameters, including main characteristics of the article (authors, year, and journal); country, aim, design, and period of the study; information-gathering technique, features of the participants (e.g., sample size, sex, age, criteria of inclusion/exclusion, comparison-group if any); main results; limitations (if any); and conclusions.

## 3. Results

**Figure 1:**
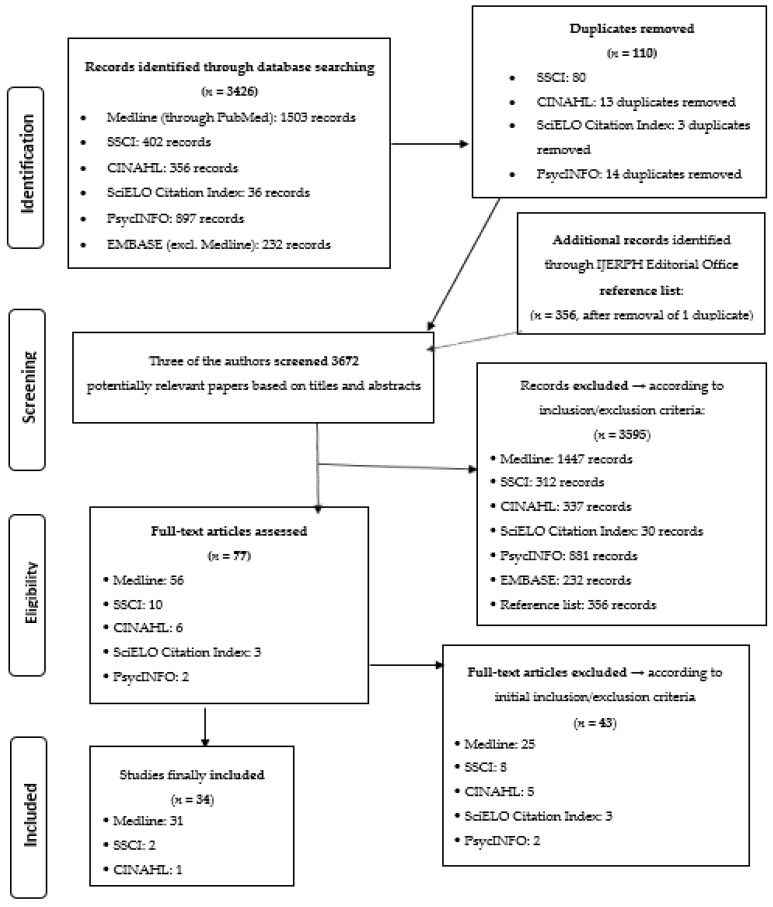
Flow diagram depicting the different phases of the scoping review about occupational health and safety of immigrant workers in Italy and Spain (1999–2018).

As shown in the flow diagram reported in Figure 1, after removing duplicates, we identified 3672 records overall. Then, we excluded most of these potentially relevant papers (*n* = 3595–98%) in the initial screening based on titles and abstracts. Overall, 77 articles were read in full, 43 of which [18,19,20,21,22,23,24,25,26,27,28,29,30,31,32,33,34,35,36,37,38,39,40,41,42,43,44,45,46,47,48,49,50,51,52,53,54,55,56,57,58,59,60] were excluded, while 34 [61,62,63,64,65,66,67,68,69,70,71,72,73,74,75,76,77,78,79,80,81,82,83,84,85,86,87,88,89,90,91,92,93,94] were finally included. 

Most (*n* = 30) of the excluded studies read in full did not report data on occupational health and safety or health-related outcomes among immigrant workers [18,19,20,22,25,27,29,30,32,33,35,36,37,38,39,41,42,43,45,46,47,48,49,50,53,54,55,57,58,60]; 11 were conference proceedings [21,24,26,31,34,40,44,51,52,56,59], and 2 literature reviews [23,28].

As summarized in Table 1, 74% of the 34 included studies were from Spain [61,62,63,64,65,66,67,68,69,70,71,72,73,74,75,76,77,78,79,80,81,82,83,84,85], 26% from Italy [86,87,88,89,90,91,92,93,94]. Most of them were quantitative [61,62,63,64,65,66,67,68,69,70,71,72,73,74,75,76,77,78,79,86,87,88,89,90,91,92,93,94], six were qualitative [80,81,82,83,84,85], 74% were cross-sectional studies [61,62,63,66,67,68,69,70,71,73,75,76,77,78,80,81,82,83,84,85,86,87,90,92,93] and nine quantitative studies were longitudinal [64,65,72,74,79,88,89,91,94]. As regards the study period, 41% of them were performed before [69,70,71,72,77,80,81,82,84,88,90,92,93,94], 47% during [61,62,63,64,67,68,73,74,75,76,78,79,83,85,87,91], and 12% (i.e. n. 4, all quantitative) after [65,66,86,89] the economic crisis. Finally, more than 80% of the studies were based on subjective data [61,62,63,64,65,66,67,68,69,70,71,73,74,75,76,77,78,79,80,81,82,83,84,85,86,87,92,93], while only six studies reported objective data [72,88,89,90,91,94].

### 3.1. Quantitative Studies

The main features of the 28 quantitative studies [61,62,63,64,65,66,67,68,69,70,71,72,73,74,75,76,77,78,79,86,87,88,89,90,91,92,93,94] are shown in Table 2 (19 cross-sectional studies) [61,62,63,66,67,68,69,70,71,73,75,76,77,78,86,87,90,92,93] and Table 3 (nine longitudinal ones) [64,65,72,74,79,88,89,91,94], respectively.

On the basis of the research question, we can distinguish the following seven main groups of studies: (i) 10 studies addressing the association of working conditions (i.e., employment conditions and/or specific work-related exposures) and health (i.e., self-reported health problems, sick leave and disability) [61,62,65,66,67,68,70,75,77,78]; (ii) two studies evaluating the association of discrimination in the workplace and health [63,92]; (iii) five studies dealing with migrant workers’ mental health [69,71,86,91,94]; (iv) one study assessing factors associated with a low risk perception of zoonoses in immigrant workers compared with natives [87]; (v) one study aimed at identifying migrant workers with hazardous drinking problem [73]; (vi) six studies addressing whether the risk of occupational injuries is higher in immigrant workers than in natives [72,76,88,89,90,93]; and (vii) three studies examining the effect of the economic crisis on migrant workers’ working conditions and on their health [64,74,79].

(i) Working conditions and health (*n* = 10 studies)

In general, compared to natives, immigrant workers in Spain showed higher prevalence of manual work or low-skilled jobs; temporary or informal employment and low wages; more exposure to physical demands; poorer (i.e. “unhealthy”, “risky”) environmental working conditions; and a greater risk of injury [66,67,70,75,77]. Therefore, immigrant workers in Spain, compared to Spanish-born workers, reported poorer self-reported general and mental health [67,68,78], worse adherence to dietary recommendations [65], and greater absence from work due to health problems [62]. In particular, undocumented foreign-born workers who lived in Spain ≤ 3 years showed the highest risk of both poor self-rated health and mental health problems [78].

It was also showed that immigrant workers had a greater sickness presenteeism as compared with Spanish-born workers, especially those who had lived in Spain for less than two years [61]. Also, the condition of immigrant decreases the probability of developing disability, probably due to better initial health status (in turn, due to the “healthy migrant effect” or to the fact that healthy people are more likely to migrate) [77].

(ii) Discrimination in the workplace and health (*n* = two studies)

In Spain, Moroccans showed the highest prevalence of perceived discrimination (answering to the question: “Have you ever felt discriminated against?”—Yes/No). Immigrant workers who felt discriminated presented a significantly higher risk of reporting both general and mental health problems [63].

In Italy, perceived discrimination (answering to the question: “Have you ever felt to be exposed to bullying or discrimination phenomena in your work environment?”—Yes/No) was higher among immigrant compared to Italian males, regardless of the geographical areas of origin, particularly for construction and other industrial workers. Among female workers, only Latin Americans and Africans had a higher occurrence of perceived discrimination as compared to Italians [92].

(iii) Mental health (*n* = five studies)

Two Spanish studies showed that immigrants who experienced bad atmosphere at work (low possibility of talking about problems and/or of being treated with respect), high quantitative and emotional demands, low development possibilities, and low support from co-workers reported worse mental health [69,71]. In particular, possible psychiatric case prevalence was higher in Ecuadorian women compared to Spanish-born ones, the main risk factors being having children, work dissatisfaction, and low salaries [69].

An Italian cross-sectional survey showed that migrant workers with high work demand perception tend to report high levels of both anxious-depressive and interpersonal disorders [86]. An Italian clinical case list reported some cases of work-related psychopathological conditions (i.e., stress and mobbing) among migrant workers, as well as some cases of fitness for work with limitations/prescriptions/recommendations due to psychiatric disorders (anxious-depressive disorders, schizophrenia, or other psychotic disorders) [91]. An Italian cohort study on patients diagnosed with a psychotic disorder showed that, compared to natives, a significantly higher number of migrants returned to work. Being occupationally active at the onset was a strong predictor of being working or studying at a 12-month follow-up [94].

(iv) Factors associated with a low risk perception of zoonoses (*n* = one study)

An Italian cross-sectional survey among workers in the agro-livestock and agro-food industry revealed significant differences in risk perception of zoonoses between immigrants and natives. Asian immigrants were the group with the highest prevalence of at risk behaviours and the lowest level of knowledge about zoonoses [87].

(v) Identification of migrant workers with hazardous drinking problem (*n* = one study)

A Spanish cross-sectional survey performed among migrants from North Africa, South America and India-Pakistan, without a comparison group, showed that the factors most closely associated with hazardous drinking were being a man, working in the construction industry or agriculture, being resident in Spain longer than seven years, and sharing a house with friends [73].

(vi) Occupational injuries (*n* = six studies) 

Two Spanish studies reported a higher risk for fatal and non-fatal occupational injury in foreign workers compared with Spanish workers, especially in industrial activities and among women [72,76].

Four Italian studies showed an overall higher risk for occupational injuries in immigrant workers compared to natives, particularly among unskilled, temporary workers from strong migratory pressure countries, working in the mechanical engineering and metallurgic sectors or in construction [88,89,90,93].

(vii) Effect of the economic crisis on working conditions and health (*n* = 3 studies)

Three Spanish studies showed that the economic crisis caused an increase in risk of poor mental health among immigrant workers, specifically those who lost their job, whose number of working hours increased, with low salaries and reporting family burden [64,74], as well as a greater risk of job loss/insecurity and of psychological and physical job demand [79].

### 3.2. Qualitative Studies

The main features of the six qualitative studies [80,81,82,83,84,85] are shown in Table 4.

These studies used individual in-depth interviews and/or focus groups to collect their data; participants were immigrant workers and/or key informants living in Spain. 

Overall, they compared immigrants and Spanish workers. Immigrants reported discrimination in their community and working life, precarious/poor working conditions, low pay, and health hazards. Exposure to occupational risks (e.g., ergonomic and psychosocial hazards) appeared to be worsened in immigrant workers, owing to their greater presence in low-skilled jobs and their economic need to prolong working hours. Immigrant workers’ health status seemed to be influenced by their working conditions; perceived discrimination seemed to affect their quality of life, with consequences on both their mental and physical health (e.g., stress, depression, sleep disturbances, diffuse muscle pain, headaches, gastric discomfort). Moreover, key informants described some difficulties in having health problems recognized as work-related, mainly because of irregular and precarious employment, employers’ and insurance companies’ reluctance, and lack of knowledge. Finally, immigrant workers in Spain experienced a deterioration in their quality of life and health during times of economic recession, which they interpreted as consequences of a worsening of employment and working conditions as well as of a significant reduction of investments in occupational health and safety measures. Moreover, in a context of economic crisis, many factors influence the occurrence of presenteeism, to which some musculoskeletal, respiratory, and mental problems appeared to be related.

With respect to migration status, two qualitative studies performed in Spain before the economic crisis reported that documentation status was relevant in terms of empowerment and bargaining but did not appear to influence work tasks or exposure to hazards directly [80]. Undocumented workers described poorer working conditions [82].

## 4. Discussion 

The main messages arising from the present scoping review are

1. Italy and Spain show similar occupational health and safety concerns and patterns of risk for fatal and non-fatal injuries; the overall numbers are impressive and demand interventions;
2. the parallel economic crisis and the irregular status of many immigrant workers contributed to exacerbate occupational health and safety concerns in Italy and Spain;
3. the uniqueness of the demographic migration process that happened in these two Mediterranean countries;
4. compared to natives, immigrant workers in Italy and Spain showed [63,64,66,67,68,69,70,71,72,74,75,76,77,78,79,80,81,82,83,84,85,86,88,89,90,91,92,93] higher prevalence of manual work or low-skilled jobs, temporary or informal employment and low wages, perceived discrimination at work; higher physical demands, poorer environmental working conditions and more exposure to occupational risks, in particular ergonomic and psychosocial hazards; worse general and mental health and employment/working conditions, mainly due to the economic crisis [78,81,82];
5. our findings are consistent with those of previous reviews investigating working conditions and occupational health outcomes among international migrant workers [7,9,11,12,13].

A few gaps can be identified, such as

1. many problems have been highlighted, but scant solutions were proposed. No intervention studies are available, which could be very useful to build an evidence-based prevention of work-related injuries and diseases among immigrant workers in the workplaces, as well as to better inform health policy makers;
2. more observational studies are needed with a longitudinal design, comparing the outcomes of interest in relationship to the different periods (i.e., before, during, and after) of the economic crisis. Such studies should be based on objective data and dealing preferably with occupational diseases instead of occupational injuries;
3. more studies should analyze the potential role played by the migration status (i.e., documented vs undocumented) in exacerbating poor health and safety outcomes among migrant workers in Italy and Spain;
4. the great majority of the included studies did not consider the heterogeneity of migrant workforce, with no sub-analyses for ethnic group and for length of stay in the host country; these are possible conditions of different vulnerability to occupational health and safety risks.

Some limitations should be acknowledged. We performed a non-systematic scoping review. Since the amount of relevant peer-reviewed literature surpassed our expectations, we did not include the grey literature (e.g., conference abstracts or proceedings, theses, dissertations, books, or book chapters). Therefore, we may have overlooked relevant documentation published. Relevant differences were observed between the studies in terms of sample size, methods of recruitment, and methods of assessing both working conditions and OH&S outcomes. This heterogeneity restricted our ability to compare and combine the findings. Therefore, the presented results are a simplification, a summary and a selection of information and knowledge available. In addition, we did not summarize the findings per migration group.

With respect to previous reviews, this paper has the following strengths: it presents updated results and comprehensive of quantitative and qualitative studies performed before, during and after the economic crisis; it deals only with migrant workers, not including refugees and asylum seekers; and, to the best of our knowledge, it is the first study specifically on two frontier Mediterranean countries.

## 5. Conclusions 

The evidence base for migrant workers’ occupational health and safety in Italy and Spain is growing considerably. The findings of this scoping review indicate the strong need for a global, response to assure that adverse occupational health and safety outcomes among migrant workers are tackled, preferably with intervention studies. Also, we believe in a strong multidisciplinary call for an equitable access to health care 

Migration represents a global and increasing challenge that needs more attention. Therefore, a concrete and evidence-based approach will be required to prevent and monitor the occupational risk factors and associated outcomes, as well as for the development of new policies and enforcement of existing ones aimed at protecting migrant workers in the workplaces. There is a need for the following key actions, from the occupational health and safety side:

1. promotion of migrant workers’ wellbeing and prevention of migrant workers’ disorders in the workplaces, by means of focused and multidisciplinary risk assessments;
2. concrete and timely responses to migrant workers’ health needs, through largely accessible and focused health surveillance, fitness for work, case management and health promotion performed by a qualified, accountable, and motivated occupational physician;
3. more collaborative dialogue with general practitioners;
4. more social protection and compensation opportunities for work-related disorders in migrant workers in general, and for female domestic workers and caregivers in particular;
5. the already existing and protecting legislation about occupational health and safety of immigrant workers should be regularly and proactively applied in the workplaces;
6. free access to public occupational health services should be provided to migrant workers, particularly undocumented ones.

The adoption of the above-suggested best practices, according to the principles of corporate social responsibility (also stated by legislation), and within the framework of a more general design of social protection and health care systems for migrant populations [95], would enable migrant workers’ effective integration, with relevant benefits for workers, enterprises, and society at large.

Finally, future research should be aimed at a deeper analysis of labor market institutions, gender, language, migrant flows, and status as mediating factors in occupational safety and health outcomes among migrant workers.

## Figures and Tables

**Table 1:** Summary of the main features of the 34 studies included in the scoping review about occupational health and safety of immigrant workers in Italy and Spain (1999–2018).

Variable	*n* (%)
Country	
Spain	25 (74%)
Italy	9 (26%)
Type of the study	
Quantitative	28 (82%)
Qualitative	6 (18%)
Design of the study	
Cross-sectional	25 (74%)
Longitudinal	9 (26%)
Study period (range 2000–2017)	
Before economic crisis (2000–2007)	14 (41%)
During economic crisis (2008–2014)	16 (47%)
After economic crisis (2015–2017)	4 (12%)
Methods of data collection	
Surveys, personal interviews, focus groups	28 (82%)
Objective data (i.e. occupational injury data, case series)	6 (18%)

**Table 2:** Main features and results of the 19 cross-sectional quantitative studies included in the scoping review about occupational health and safety of immigrant workers in Italy and Spain (1999–2018).

Author (Ref Number), Country, Study Period	Aim/Objectives	Method, Sample	Main Results
Before economic crisis (2000–2007)			
Del Amo et al. [69], Spain, 2006–2007	To describe the prevalence of and the risk factors for poor mental health in female and male Ecuadorian migrants in Spain.	• Cross-sectional survey • Personal interviews (questionnaire-based) • 1122 people: ✓ 554 natives; ✓ 568 Ecuadorian immigrants	Possible psychiatric case (PPC, measured as score of ≥5 on the General Health Questionnaire-28) prevalence was higher in Ecuadorian (34%, 95% CI 29–40%) and Spanish women (24%, 95% CI 19–29%) compared to Ecuadorian (14%, 95% CI 10–18%) and Spanish men (12%, 95% CI 8–16%). Shared risk factors for PPC between Spanish and Ecuadorian women were: having children (OR 3.1, 95% CI 1.4–6.9), work dissatisfaction (OR 4.1, 95% CI 1.6–10.5), low salaries (OR 2.5, 95% CI 1.1–5.9), no economic support (OR 1.8, 95% CI 0.9–3.4), and no friends (OR 2.2, 95% CI 1.1–4.2). Shared risk factors for PPC in Ecuadorian and Spanish men were: bad atmosphere at work (OR 2.4, 95% CI 1.3–4.4), no economic support (OR 3.5, 95% CI 1.3–9.5), no friends (OR 2.5, 95% CI 0.9–6.6), and low social support (OR 1.6, 95% CI 0.9–2.9).
Diaz-Serrano et al. [70], Spain, 2006	To determine which of the working conditions are perceived as (dis)amenities by the different groups of workers.	• Cross-sectional **survey** • (2006 Health Survey of Catalonia) ✓ 7940 adults (16-65 years old) ✓ 6580 Catalan-born ✓ 910 immigrants (380 Latin America, 260 African, 150 European Union, 120 other countries).	Immigrants, particularly the African sub-group, are more tolerant of jobs involving poorer environmental working conditions (e.g., risk of injury, mean 0.17 vs. 0.12; work too much, mean 2.23 vs. 2.16), more physically demanding tasks, and higher exposure to physical damage (e.g., exposure to noise, mean 1.77 vs. 1.65; exposure to dust, mean 1.86 vs. 1.59; move heavy loads, mean 1.78 vs. 1.56; repetitive movements, mean 2.54 vs. 2.33). Immigrant workers, particularly the Latin-American sub-group, tend to be employed in lower quality jobs and to enjoy worse contractual conditions than natives (e.g., having permanent contract, mean 0.47 vs. 0.74; without contract, mean 0.09 vs. 0.03).
Font et al. [71], Spain, 2004-2005	To examine the relationship between immigration and mental health, taking into account the psychosocial factors in the workplace.	• Cross-sectional survey. • Personal interviews, (questionnaire-based). • 7555 workers: ✓ 6868 natives; ✓ 687 immigrants.	Immigrants who experienced high quantitative demands (PR = 1.46; CI 95%:1.34–1.59), high emotional demands (PR = 1.42; CI 95%:1.30–1.56), high demands for hiding emotions (PR = 1.35; CI 95%:1.21–1.50), low possibilities for development (PR = 1.21; CI 95%:1.09–1.33), low levels of support from coworkers (PR = 1.41; CI 95%:1.30–1.53), and low esteem (PR = 1.53; CI 95%:1.42–1.66) perceived worse mental health. Immigrants with a high influence (PR = 1.19; CI 95%:1.09–1.29) and high control over working times (PR = 1.25; CI 95%:1.14–1.36) also reported worse mental health.
Solé et al. [77], Spain, 2006	To assess disparities between immigrants and natives in the role played by working conditions in determining the occurrence of disability.	• Cross-sectional analysis of data from Continuous Sample of Working Lives 2006 (provided by Social Security administration). • 718,958 working-age individuals (21–64 years old): ✓ 681,078 Spaniards; ✓ 37,880 immigrants (from Africa, Latin America, Europe, USA, Canada, and Asia).	The proportion of immigrants on temporary contracts or low-skilled jobs is much higher than that of natives (47.73% vs. 37.31% and 35.19% vs. 28.14% respectively). Immigrants are also more likely to be employed in high-risk jobs; the difference is not large (27.4% vs. 26.8%), but it is statistically significant. When the three potentially “unhealthy” working conditions are jointly considered, the proportion of immigrants employed in temporary, low-skilled, or high-risk positions is nearly twice that of native-born Spaniards. However, being an immigrant reduces the probability of disability by nearly 0.9%. Better initial health status (i.e. the so-called “healthy migrant effect” or else healthy people are the more likely ones to migrate) could explain this result.
Patussi et al. [90], Italy, 2003	To evaluate the difference in the frequency of occupational injuries between between permanent and temporary workers and between Italian and immigrant workers.	• Cross-sectional comparison of incidence rate of occupational injury. • Setting: 160 factories and four employment agencies operating in Friuli-Venezia Giulia, Italy. • Participants: ✓ 18,210 permanent workers; ✓ 1345 temporary workers.	There were 1499 occupational injuries among permanent workers and 392 among temporary workers. Nationality appears to be an important risk factor among permanent workers in general (IR 1.63; 95% CI 1.34–1.98), especially in the wood industry (IR 1.39; 95% CI 1.04–1.87) and the metal working sector (IR 1.82; 95% CI 1.53–2.15). Nationality appears to be a significant risk factor among temporary workers only in the metal working sector (IR 1.44; 95% CI 1.03–2.02). The incidence rate ratio of occupational injury was significantly higher in temporary workers than in permanent workers (IR 2.46; 95% CI 2.02–2.99).
Salvatore et al. [92], Italy, 2007	To compare the occurrence of episodes of arrogance or discrimination perceived at the workplace between documented immigrants and Italians.	Analysis of data from the 2007 cross-sectional Labour Force Survey (questionnaire-based) conducted by the Italian National Institute of Statistics. 61,214 workers, of which 2203 immigrants.	The occurrence of perceived arrogance or discrimination was higher among immigrant compared to Italian males for all geographical areas of origin considered. Adjusted ORs were 4.6 (95% CI:3.6–5.8) for Africans, 3.4 (95% CI:2.5–4.6) for Asians, 2.1 (95% CI:1.6–2.8) for Eastern Europeans, and 2.0 (95% CI:1.0–3.7) for Latin Americans. Among female workers only Latin Americans and Africans showed a higher occurrence of perceived arrogance or discrimination compared to Italians: adjusted ORs were respectively 3.9 (95% CI:2.6–5.7) and 2.6 (95% CI:1.5–4.5).
Salvatore et al. [93], Italy, 2007	To compare the occurrence of work-related injuries between legally residing immigrants and Italians.	Analysis of data from the 2007 cross-sectional Labour Force Survey (questionnaire-based) conducted by the Italian National Institute of Statistics. 61,214 workers, of which 2203 were immigrants.	The occurrence of work-related injuries was significantly higher among immigrant males compared to Italian males (adjusted OR = 1.82; 95% CI 1.53–2.16). In particular, for construction workers, the odds of injury were twice as high for immigrant men compared to Italians (adjusted OR = 2.05; 95% CI 1.56–2.69). For unskilled construction workers, the odds of injury were nearly nine times higher for immigrant men (OR = 8.64; 95% CI 2.85–26.20).
**During economic crisis (2008–2014)**			
Agudelo-Suárez et al. [61], Spain, 2008–2009	To assess the extent of sickness presenteeism in a sample of Spanish-born and foreign-born workers.	Cross-sectional questionnaire-based survey. 1617 foreign-born workers (from Colombia, Ecuador, Morocco and Romania; who had been living in Spain for at least one year; who had been working in Spain for at least three months). 442 Spanish-born workers (resembling the foreign-born sample according to gender, age 20–40 years old, and area of residence in Spain).	Foreign-born workers were more likely to report sickness presenteeism compared with their Spanish-born counterparts (Prevalence: 42% in Spanish-born and 56.3% in Foreign-born; aOR 1.77 95% CI 1.24–2.53). Among foreign-born workers, men (aOR 2.31; 95% CI 1.40–3.80), those with university studies (aOR 3.01; 95% CI 1.04–8.69), temporary contracts (aOR 2.26 95%; CI 1.29–3.98), and salaries between €751–1200 per month (aOR 1.74; 95% CI 1.04–2.92) were more likely to report sickness presenteeism.
Agudelo-Suárez et al. [62], Spain, 2008	To describe the migratory process (reasons for migrating, time of residence), legal status and personal, working and health characteristics of immigrants with work experience in Spain.	Cross-sectional questionnaire-based survey. 2434 immigrant workers (Colombia, Ecuador, Morocco and Romania).	The immigrants were working in jobs that were below their educational level and reported problems concerning the type of contract, salaries, and the length of the working week, which was often more than 40 hours. They frequently reported general health problems (18%), mental health problems (27%), absence from work due to health problems (48%), and occupational injuries requiring medical care (23%).
Agudelo-Suárez et al. [63], Spain, 2008	To analyze the relationship between immigrants’ perceived discrimination and various self-reported health indicators.	Cross-sectional questionnaire-based survey. 2434 immigrant workers (Colombia, Ecuador, Morocco and Romania).	75.4% of participants reported at least one type of discrimination. Moroccans showed the highest prevalence of perceived discrimination. Immigrants reporting discrimination were at significantly higher risk of reporting health problems. Workplace-related discrimination was associated with poor mental health (aOR 2.97; 95% CI 2.45-3.60), insomnia (aOR 2.06; 95% CI 1.64–2.60), anxiety (aOR 1.79; 95% CI 1.44–2.23), muscular problems (aOR 1.70; 95% CI 1.41–2.04), headache (aOR 1.68; 95% CI 1.41–2.00), and the worsening of self-rated health (aOR 2.20; 95%CI 1.73–2.80).
Cayuela et al. [67], Spain, 2011-2012	To examine differences between workers related to migrant-status, self-perceived and mental health.	• Cross-sectional survey. • Personal interviews (questionnaire-based). • 8591 workers: ✓ 711 immigrants; ✓ 7880 Spaniards.	Immigrants reported more exposure to physical demands (38.3 vs. 24.3% men; 31.3 vs. 13.7% women) and higher prevalence of temporary or no contract than natives. Mental (OR 2.02; CI 1.39–2.93) and self-perceived health (OR 2.64; CI 1.77–3.93) were poorer for settled immigrant women compared to natives. Job satisfaction accounted for 15.8% of the difference in self-perceived health.
Conway et al. [68], Spain, 2011	To assess the relationship between long work hours (LWH) and self-reported general health (SRGH).	• Cross-sectional analysis of data from 2011 Spanish National Survey of Working Conditions (VII-ENCT). • 8306 workers: ✓ 7967 natives; ✓ 339 Latin American immigrants.	Immigrant workers were at approximately twofold increased odds of reporting poor SRGH compared to their native counterparts (OR = 1.86; 95%CI = 1.43–2.41). Natives working >51 h per week had increased odds of reporting poor SRGH compared to those working fewer hours (OR = 2.17; 95% CI = 1.71–2.75). LWH were associated with differential health outcomes in populations of native and Latin American immigrant workers in Spain, which may reflect social or occupational inequalities in general or resulting from the 2008 financial crisis.
Perez-Carceles et al. [73], Spain, 2010–2012	To identify migrant workers with a hazardous drinking problem by means of a self-reported questionnaire and a biomarker and to ascertain associated risk factors.	Cross-sectional survey. 385 migrant workers (from North Africa, South America, and India-Pakistan).	13.8% (*n* = 53) of the workers were screened as positive with the Alcohol Use Disorders Identification Test - AUDIT (≥8) and/or Carbohydrate-Deficient Transferrin test - CDT (>2.6) and identified as hazardous drinkers. 70% of the South Americans taking part drank alcohol compared with the 69% of North Africans who were tee-totallers, mainly as a consequence of their Islamic religion. Despite this, 30% of the North Africans workers consumed alcohol and 16% can be considered hazardous drinkers. The highest percentage of hazardous drinkers worked in the construction industry (17%), and the lowest percentage of hazardous drinkers worked in the service sector (7%). Being a man (OR: 2.0), working in the construction industry (OR: 2.8) or agriculture (OR: 2.2), being resident in Spain for more than seven years (OR: 2.3) and sharing a house with friends (OR: 3.1) were the factors most closely associated with hazardous drinking.
Ronda et al. [75], Spain, 2008	To compare self-reported exposure to occupational health risks in foreign-born and Spanish-born workers in Spain.	Cross-sectional survey. Face-to-face interviews (questionnaire-based). 1841 foreign-born workers (Morocco, Ecuador, Romania, Colombia). 509 Spanish workers.	Foreign-born men were employed mainly in manual jobs (75.4 %) and frequently held temporary contracts, while nearly 30% of them had no contract. The prevalence of self-reported exposure to occupational health risks for foreign-born workers of both sexes was significantly higher than Spanish-born workers for working many hours standing up (55.2% vs. 46.6%, *p* < 0.05), working with extreme temperatures up (27.5% vs 15.6%, *p* < 0.05), and working many hours/day up (39.9% vs. 29.4%, *p* < 0.05). Foreign-born female manual workers were more likely than Spanish workers to report working many hours/day (aOR 2.68; 95% CI 1.06–6.78).
Rubiales-Gutiérrez et al. [76], Spain, 2008	To compare the occupational accidents between autochthonous and immigrant workers in Spain.	• Cross-sectional analysis of data from 2008 Spanish National Survey of Working Conditions (VI-ENCT). • 10,927 workers: ✓ 9584 Spaniards; ✓ 1343 immigrants (from low Human Development Index (HDI) countries).	The prevalence of temporary contracts was higher among workers from low HDI countries compared to Spaniards (15.4% vs. 9.7%; adjusted OR 1.64; 95% CI 1.22–2.20). The prevalence of occupational accidents was 12.7% (women, 11.1%) for workers from low HDI countries and 10.3% (women, 8.1%) for Spaniards. A higher risk of occupational accidents was observed among workers from low HDI countries compared to Spaniards (adjusted OR 1.36; IC 95% 1.12–1.65), especially among women (adjusted OR 1.66; 95% CI 1.21–2.28).
Sousa et al. [78], Spain, 2008-2009	To analyze the relationship of legal status and employment conditions with health indicators in foreign-born and Spanish-born workers in Spain.	• Cross-sectional survey. • Personal interviews (questionnaire-based). • 2358 workers: ✓ 1849 immigrants (from Morocco, Ecuador, Romania and Colombia); ✓ 509 Spanish-born.	Among females, the highest risks of poor self-rated health were observed in foreign-born workers (time in Spain >3 years) without contracts (aOR 4.63; 95% CI 1.95–10.97) and with temporary contracts (aOR 2.36; CI 95% 1.13–4.91). In males, undocumented foreign-born workers who lived in Spain ≤ 3 years (aOR 2.26; CI 95% 1.15–4.42) and foreign-born workers who lived >3 years and worked with temporary contracts (aOR 1.96; 95% CI 1.13–3.38) experienced the highest risk of mental health problems, relative to their Spanish-born permanently contracted counterparts. With respect to poor self-rated health, undocumented foreign-born workers who lived in Spain ≤ 3 years (aOR 2.68; CI 95% 1.09–6.56) and Spanish-born temporarily contracted workers (aOR 2.40; 95% CI 1.04–5.56) were at the highest risk.
Cediel et al. [87], Italy, 2008-2009	To assess factors associated with a low risk perception of zoonoses in immigrant and Italian workers.	• Cross-sectional survey (questionnaire-based). • 175 workers in the agro-livestock and agro-food industry: ✓ 93 immigrants (from Romania, Marocco, Albania, India, China, Argentina, Perù, Macedonia, Ivory Coast, Ukraine, and Colombia); ✓ 82 Italians.	The study revealed significant differences in risk perception at work (*p* = 0.001). Associations were found between “not having correct knowledge about zoonoses” and the following variables: i. “being immigrant” OR = 4.1 (95% CI 1.7; 9.8 *p* ≤ 0.01); ii. “working in the livestock industry” OR = 2.9 (95% CI 1.2; 15.4 *p* = 0.01); and iii. “being an unqualified worker” OR = 4.4 (95% CI 2.9; 15.4 *p* ≤ 0.01). Another strong association was found between being immigrant and having a low job qualification OR = 6.7 (IC 95%; 2.9–15.4 *p* ≤ 0.01). Asian immigrants were the group with the highest frequency of risky behaviours and the lowest level of knowledge about zoonoses.
**After economic crisis (2015–2017)**			
Cayuela et al. [66], Spain, 2015	To analyze the relationship between working hours (WHs) and the likelihood of poor self-reported general health (SRGH).	• Cross-sectional analyses from a prospective cohort study. • Survey/Personal interviews (questionnaire-based). • 306 adult workers: ✓ 217 immigrants (Colombia, Ecuador) ✓ 89 Spaniards.	Immigrant single-parent families were more likely to report poor SRGH for three WH categories: 20 WH/week (prevalence ratio [PR] = 3.3, 95% confidence interval [CI] 1.6–7.2), >30–40 WH/week (PR = 2.8, 95% CI 1.3-6.4), and >40 WH/week (PR = 4.2, 95% CI 1.8–10.1). Highest prevalence of poor SRGH (72.7%) was reported by immigrant, single-parent workers working >40 WH/week. A 5-h difference in the WHs was found between single-parent immigrant workers and two-parent immigrant workers, which highlights the possible role of family structure as a moderator of the influence of adverse job conditions, including long working hours.
Capasso et al. [86], Italy, 2015	To test a multi-dimensional model in the prediction of subjectives reports of health by workers differing in ethnicity.	• Cross-sectional survey (questionnaire-based). • 900 workers: ✓ 250 Eastern European care workers; ✓ 250 Moroccan factory workers; ✓ 200 Ghanaian masons; ✓ 100 Italian factory workers; ✓ 100 Italian masons.	60.6% (*n* = 272) and 62.1% (*n* = 278) of migrant workers with high perception of work demands reported respectively high levels of anxious-depressive disorders and high levels of interpersonal disorders. 51.5% (*n* = 282) and 53.3% (*n* = 300) of migrant workers who had experienced racial discrimination suffered respectively high levels of anxious-depressive disorders and high levels of interpersonal disorders. Moroccan and Ghanaian workers may be more likely to suffer interpersonal disorders (OR = 3.230; CI = 1.589–6.565), (OR = 3.134; CI = 1.507–6.518). Eastern European care workers (OR = 2.955; CI = 1.573–5.552) as well as the immigrant workers who had experienced racial discrimination (OR = 2.422; CI = 1.344–4.856) may be more likely to suffer anxious-depressive disorders.

IR = Incidence Ratio; OR = Odds Ratio; PR = Prevalence Ratio.

**Table 3:** Main features and results of the nine longitudinal quantitative studies included in the scoping review about occupational health and safety of immigrant workers in Italy and Spain (1999–2018).

Author (Ref Number), Country, Study Period	Aim/Objectives	Method, Sample	Main Results
Before economic crisis (2000–2007)			
López-Jacob et al. [72], Spain, 2005	To compare the incidences for both fatal and non-fatal injuries in foreign workers to that of Spanish workers.	Longitudinal analysis on injury data (from the accident registry of the ministry of labor and social issues).	Overall, relative risk for occupational injury in foreign workers in 2005 was superior to base risk for both fatal (RR 1.34; 95% CI: 1.11–1.62) and non-fatal injury (RR 1.13; 95% CI: 1.13–1.14). Compared with Spanish workers, risk for both fatal and non-fatal occupational injury was higher for foreign workers in industrial activities (non-fatal = RR 2.03; 95% CI: 1.89–2.18; fatal = RR 4.53; 95% CI: 1.37–14.96), while it was lower in construction (non-fatal = RR 0.79; 95% CI: 0.78–0.80; fatal = RR 1.04; 95% CI: 0.80–1.37) and commerce/restaurants/hotels (non-fatal = RR 0.92; 95% CI: 0.91–0.94; fatal = RR 0.75; 95% CI: 0.42–1.31).
Colao et al. [88], Italy, 2000–2003	To describe the trend of work accidents in the Local Health Area of Fabriano (Marche Region), during the period 2000–2003.	Longitudinal analysis on injury data (from “New Informative Flows” database set up by Italian National Institute of Insurance for Occupational Injury).	Occupational accidents among immigrant workers gradually rose and peaked in 2002. The sectors with high rates of accidents were the mechanical engineering and metallurgic sectors and the construction industry. Accidents occurred mainly among young people (18 to 34 years old), and there was a prevalence of men (83.3%). The number of occupational accidents with a prognosis of 8–30 days fell progressively for workers in general but gradually rose for immigrant workers with a peak in 2001. The overall number of occupational accidents that caused permanent invalidity fell by 52.3% for the workforce in general, and by 25% among immigrant workers.
Tarricone et al. [94], Italy, 2002–2009	To evaluate the occupational outcomes of a first episode psychosis (FEP) sample in Bologna (Northern Italy).	• Longitudinal cohort study. • 12-month follow-up of all FEP patients (18-64 years) identified in the study period. • 163 patients: ✓ 24% (*n* = 39) first generation migrants (from Europe, Asia, North-Africa, Sub-Saharan Africa, and Central-South America).	Migrants from European countries as well as migrants from non- European countries suspended work or study activity significantly more often than natives (14, 58% vs 22, 31.8%; adjusted OR 2.88, 95% CI 1.26–6.57). A significantly higher percentage of migrants returned to work compared to natives (9, 64% vs. 8, 40%; adjusted OR 4.45, 95% CI 1.55–12.76). Those who had a diagnosis of schizophrenia were more frequently inactive at 12months from the onset than patients with other psychotic disorders (34, 56% vs. 32, 40%, c sq = 3.4, *p* = 0.06); the odds ratio was significant after adjusting for age and gender and migrant status (OR 2.54, 95% CI 1.05–6.13). To be occupationally active at the onset was a strong predictor of being working or studying at 12 months follow-up, with an OR 20-time greater compared to this of people inactive at the onset.
During economic crisis (2008–2014)			
Agudelo-Suárez et al. [64], Spain, 2008 and 2011	To assess changes in mental health in a sample of migrant workers after the eruption of the economic crisis in Spain.	• Longitudina**l** study. • Survey/Personal interviews (questionnaire-based): ✓ 2008 = face-to-face ✓ 2011 = phone • 318 immigrant workers (Colombia, Ecuador, Morocco and Romania).	Change in prevalence of poor mental health was higher in men (aOR 4.63; 95% CI 2.11–10.16); specifically those unemployed (aOR 8.34; 95% CI 2.19–31.75), with low salaries <1,200 euros (aOR 5.15; 95% CI 2.07–12.80), and those reporting family burden (aOR 4.01; 95% CI 1.73–9.26). An increase of the OR in 2011 with respect to 2008 was found in unemployed women and those with familiar burden, although without significant associations.
Robert et al. [74], Spain, 2008 and 2011	To evaluate the influence of changes in employment conditions on the incidence of poor mental health of immigrant workers in Spain, after a period of 3 years, in context of economic crisis.	• Longitudinal survey. • Survey/Personal interviews (questionnaire-based): ✓ 2008 = face-to-face; ✓ 2011 = phone. • 214 immigrant workers (Colombia, Ecuador, Morocco and Romania).	There was an increased risk of poor mental health in workers who lost their jobs (aOR = 3.62, 95%CI: 1.64–7.96), whose number of working hours increased (aOR = 2.35, 95%CI: 1.02–5.44), whose monthly income decreased (aOR = 2.75, 95%CI: 1.08–7.00) or who remained within the low-income bracket. This was also the case for people whose legal status (permission for working and residing in Spain) was temporary or permanent compared with those with Spanish nationality (aOR = 3.32, 95%CI: 1.15–9.58) or illegal (aOR = 17.34, 95%CI: 1.96–153.23).
Torá et al. [79], Spain, 2007 and 2011	To assess whether the prevalence of adverse psychosocial working conditions changed for Spanish and foreign workers between 2007 (pre-economic recession) and 2011 (post-economic recession).	• Longitudinal analysis based on the sixth and seventh editions of the cross-sectional Spanish Survey of Working Conditions. • Workers employed at the time of the interview: ✓ *n* = 10402 in 2007; 1322 foreign-born and 9080 Spanish; ✓ *n* = 8438 in 2011; 786 foreign-born and 7652 Spanish.	Foreign workers made up a smaller proportion of the workforce in Spain in 2011 compared to 2007 (9.3% and 12.7%, respectively). There was no significant difference in psychological job demands in foreign workers between 2007 and 2011. Both Spanish and foreign national workers reported decreased physical demands in 2011. Among both Spanish and foreign national workers, greater aPR (adjusted prevalence ratios) were found for job loss/insecurity in 2011 compared to 2007 (aPR = 2.47; 95% CI = (2.34–2.60); aPR = 2.44; 95% CI = (2.15–2.77), respectively).
Porru et al. [91], Italy, 2001–2013	To present data from a clinical case list of workers assessed for psychosocial issues at a Northern Italy public occupational health unit.	Longitudinal case series. 20 migrant workers (from Albania, Morocco, Serbia, Pakistan, Senegal, Egypt, Ethiopia, Romania, and Togo).	They were mainly employed in metal (35%) and manufacturing (15%) industry, followed by health (20%), catering/hospitality (15%), metal casting (10%), and transport (5%) sectors. They were sent by general practitioners (70%) and enterprises (30%). Some limitations/prescriptions/recommendations were finally expressed in some cases in order to manage particular situations. Four (20% of assessed cases) work-related psychopathological conditions were diagnosed and notified.
After economic crisis (2015–2017)			
Benazizi et al. [65], Spain, 2015–2017	To analyze the influence of employment conditions on adherence to dietary recommendations.	• Longitudinal cohort study. • Survey/Personal interviews (questionnaire-based): ✓ 2015 = baseline ✓ 2016 and 2017 = two follow-up waves. • 215 adult workers: ✓ 148 immigrants (Colombia, Ecuador) ✓ 67 Spaniards.	Adherence to dietary recommendations was greater among Spaniards, followed by immigrants with >14 years of residence and <14 years of residence. The greatest adherence among Spaniards was for eggs (immigrants ≥14 years: 1/ORa = 2.89, <14 years: 1/ORa = 3.92), fish (immigrants ≥ 14 immigrants: 1/ORa = 2.33, <14 years: 1/ORa = 4.72), vegetables (immigrants ≥ 14 years: 1/ORa = 3.26, <14 years: 1/ORa = 4.87), dairy products (immigrants ≥ 14 years: 1/ORa = 14.34, <14 years: 1/ORa = 26.78), and sugary drinks (immigrants ≥ 14 years: 1/ORa = 2.12, <14 years: 1/ORa = 3.48), and the lowest adherence was for the consumption of sausages and cold cuts (immigrants ≥ 14 years: Ora = 7.62, <14 years: ORa = 24.65).
Giraudo et al. [89], Italy, 2017	To study the injury risk differentials between migrants and natives.	Longitudinal analysis on injury data (from the Work History Italian Panel-Salute integrated database).	Injury rates among workers from strong migratory pressure countries were higher than for workers from high income countries in engineering (15.61 ‰ py vs. 8.92 ‰ py), but there were no significant differences in construction (11.21 vs. 10.09), transportation and storage (7.82 vs. 7.23) and the wholesale and retail sectors (4.06 vs. 4.67). Injury rates for Moroccans were higher than for both HIC and total migrant workers in all economic sectors considered (IRR: 2.17 in engineering, 1.42 in construction, 2.11 in wholesale and retail trade, 1.43 in transportation and storage). The multivariate analysis revealed an interaction effect of job tenure: both total migrant workers (IRR 1.16; 95% CI: 1.01–1.34) and Moroccans separately (IRR 1.56; 95% CI: 1.33–1.82) have higher occupational injury rates than Italians in the engineering and construction sectors, after two years of experience within the job.

RR = Relative Risk or Risk Ratio.

**Table 4:** Main features and results of the six qualitative studies included in the scoping review about occupational health and safety of immigrant workers in Italy and Spain (1999–2018).

Author (Ref Number), Country, Study Period	Aim/Objectives	Method, Sample	Main Results
Before economic crisis (2000–2007)			
Agudelo-Suárez et al. [80], Spain, 2006–2007	To research perceptions of discrimination and the specific relationship between discrimination in the workplace and health among Spain’s immigrant population.	Cross-sectional interviews (*n* = 84) and focus groups (*n* = 12). Immigrant workers (from Morocco, Romania, Colombia, Sub-Saharan Africa, and Ecuador).	Participants reported instances of discrimination in their community and working life, characterized by experiences of mistreatment and precarious working conditions. Their health status seemed to be influenced by their working conditions. Perceived discrimination affected their quality of life, with consequences such as stress and other mental health problems.
Ahonen et al. [81], Spain, 2006–2007	To examine the environmental, ergonomic, and psychosocial hazards and health effects identified by immigrant women working in household service in five Spanish cities.	Cross-sectional focus groups and semi-structured individual interviews. 46 documented and undocumented immigrant women (from Colombia, Morocco, Senegal, Romania, and Ecuador) in household services.	Informants reported a number of environmental, ergonomic and psychosocial hazards and corresponding health effects. Psychosocial hazards were especially strongly present. Data on reported hazards were similar by documentation status and varied by several emerging categories: whether participants were primarily cleaners or carers, and whether they lived in or outside of the homes of their employers. Documentation status was relevant in terms of empowerment and bargaining but did not appear to influence work tasks or exposure to hazards directly.
Ahonen et al. [82], Spain, 2006–2007	To explore the perceptions that immigrant workers in Spain had of their working conditions.	Cross-sectional semi-structured focus groups and individual interviews. 158 immigrant workers (from Colombia, Morocco, sub-Saharan Africa, Romania, and Ecuador), documented or undocumented.	Participants described poor working conditions, low pay, and health hazards. Undocumented workers described poorer conditions. Documented participants also felt vulnerable because of their immigrant status. Informants believed that deficient language skills, non-transferability of their education and training, and, most of all, their immigrant status and economic need left them with little choice but to work under poor conditions.
García et al. [84], Spain, 2006	To describe the characteristics, working conditions, and occupational health situation of immigrant workers in Spain through key informants.	Cross-sectional in-depth interviews. 43 key informants (from Latin America, Africa, extra-EU countries).	Informants described difficulties in having health problems recognized as work-related, due to irregular and precarious employment, employers’ and insurance companies’ reluctance, and immigrants’ lack of knowledge. Exposure to occupational risks was exacerbated in immigrants because of their greater presence in unqualified jobs and their economic need to prolong working hours. Immigrants had little knowledge of their occupational health and safety related rights.
During economic crisis (2008–2014)			
Galon et al. [83], Spain, 2012	To explore perceptions about the factors that lead to presenteeism in immigrant workers considering the context of economic crisis.	• Cross-sectional focus group discussions. • 44 immigrant workers: ✓ 22 men, 22 women ✓ from Colombia, Morocco, Ecuador ✓ employed in different sectors.	Four categories were identified as factors that influence the occurrence of presenteeism in a context of economic crisis—poor employment conditions, fear of unemployment, employer/employee relationship, and difficulties in finding temporary replacement workers. Musculoskeletal, respiratory, and mental problems were related to presenteeism.
Ronda et al. [85], Spain, 2012	To analyse how immigrant workers in Spain experienced changes in their working and employment conditions brought about Spain’s economic recession and the impact of these changes on their living conditions and health status.	• Cross-sectional ✓ focus group discussions with 44 immigrant workers (from Colombia, Ecuador, and Morocco) ✓ individual interviews with two key informants (from Romania).	Participants experienced: a reduction in employment opportunities brought about by the crisis; a significant and negative effect of the economic recession on business investment in health and safety measures; a deterioration in their quality of life, that they attributed to the worsening of working conditions; stress, depression, sleep disturbances and impairment of their physical health (e.g. diffuse muscle pain, headaches, gastric discomfort).
